# Complementary pharmacokinetic measures to further define the profile of once-daily OROS hydromorphone ER during single-dose and steady-state dosing

**DOI:** 10.1186/2193-1801-2-625

**Published:** 2013-11-21

**Authors:** Krishna Devarakonda, Joris Vandenbossche, Ute Richarz

**Affiliations:** Clinical Pharmacology & Pharmacokinetics, Mallinckrodt Inc., 325 James McDonnell Blvd 302W-3, Hazelwood, MO 63042 USA; Clinical Pharmacology, Janssen Research & Development, Beerse, Belgium; Global Medical Affairs Lead, Janssen Global Services, Zug, Switzerland

**Keywords:** Pharmacokinetics, Hydromorphone, Extended-release, Opioids, Chronic pain

## Abstract

Conventional measures such as maximum plasma concentration (*C*_*max*_) and area under the concentration versus time curve (*AUC*) may be insufficient to fully describe the pharmacokinetic (PK) profile of extended-release (ER) formulations. A complementary measure, the half-value duration (*HVD*), corresponds to the period of time during a dosing cycle that plasma concentration is at or above half the value of the maximal concentration (i.e. ≥50% *C*_*max*_). The current post-hoc analysis uses data from 2 previously published studies comparing the PK profiles and *HVD* of OROS hydromorphone ER (16 mg administered once daily) and immediate-release (IR) hydromorphone (4 mg administered every 6 hours), calculating single-dose and steady-state condition values. Bioequivalence was demonstrated between the 2 formulations. Mean steady-state once-daily OROS hydromorphone ER concentrations were elevated for most of the 24-hour dosing period and for significantly longer than with the dose-equivalent IR hydromorphone regimen. The duration of time spent ≥50% *C*_*max*_ was, on average, 2.7 times longer at steady state for the ER formulation, which also maintained steady-state hydromorphone plasma concentrations, with 65% lower mean degree of fluctuation versus IR hydromorphone. Both formulations appeared to be well tolerated.

**Electronic supplementary material:**

The online version of this article (doi:10.1186/2193-1801-2-625) contains supplementary material, which is available to authorized users.

## Introduction

Hydromorphone, an opioid analgesic introduced into clinical practice in the 1920s, has been used extensively to treat pain (Quigley and Wiffen 2003). The pharmacokinetic (PK) profile of OROS hydromorphone extended-release (ER) is well established in healthy subjects, with controlled release resulting in sustained plasma concentrations (Angst et al. 2001; Drover et al. 2002; Moore et al. 2010; Moore et al. 2011; Sathyan et al. 2007; Sathyan et al. 2008). The hydromorphone release rate is independent of pH and gastric motility (Gupta and Sathyan 2007), and relatively unaffected by alcohol, rendering “dose dumping” unlikely (Sathyan et al. 2008). OROS hydromorphone ER reaches maximum plasma concentration (*C*_*max*_) 12 to 16 hours after administration and produces sustained hydromorphone concentrations over 24 hours, with dose-proportional PK (Drover et al. 2002; Sathyan et al. 2007; Gupta and Sathyan 2007). OROS hydromorphone ER has a long half-life (~13 to 15 hours) and requires 3 to 4 days to reach steady-state plasma hydromorphone concentrations (Moore et al. 2010; Moore et al. 2011; Sathyan et al. 2007; Gupta and Sathyan 2007).

Though PK measures such as *C*_*max*_ and area under the concentration versus time curve (*AUC*) describe properties of conventional opioid formulations, they may be insufficient or misleading for modified-release formulations. PK parameters must quantify the constant rate of controlled release in opioids and facilitate comparisons between formulations with respect to rate and extent of absorption (i.e. bioavailability/bioequivalence) (Steinijans 1990). The degree of peak-to-trough fluctuation is one metric for evaluating modified-release dosing regimens, including ER opioids. These fluctuations are lessened with OROS hydromorphone ER (mean, 60.5%), compared with immediate-release (IR) hydromorphone (172%) (Moore et al. 2010).

A complementary measure of modified-release drug performance, “plateau time”—the period of time during a dosing cycle over which plasma concentration deviates from the maximum by less than a specified percentage (Steinijans 1990)—has been applied to calibrate the performance of multiple ER formulations (Steinijans 1990; Bialer et al. 1998; Drewe et al. 1992). Various percentage thresholds have been proposed as clinically relevant correlates of the width of the efficacy range for ER drugs, including the duration of plasma concentrations at or above half the value of the maximal concentration (i.e. ≥50% *C*_*max*_, also referred to as “half-value duration” [*HVD*]) (Steinijans 1990; Bialer et al. 1998; Drewe et al. 1992; Meier et al. 1974; Guttler 2012). Other thresholds (e.g. ≥75% or ≥80%) have also been used (Steinijans 1990; Meier et al. 1974). In an experimental pain model using OROS hydromorphone ER, hydromorphone concentration peaked significantly later (median, 12 vs. 0.8 hours) and remained ≥50% *C*_*max*_ substantially longer (mean, 22.7 hours) than concentrations following a single dose of IR hydromorphone (1.1 hours). Analgesic effects also peaked significantly later (9.0 vs. 1.5 hours) and were maintained significantly longer at >50% peak analgesic effect (13.3 vs. 3.6 hours). There was a significant linear relationship (*P* < 0.05) between hydromorphone plasma concentration and analgesia (Angst et al. 2001).

The current analysis used data from 2 previously published studies in healthy subjects: one assessing effects of food on the PK of single doses of the OROS formulation compared with 4 doses of IR hydromorphone (Study A) (Moore et al. 2011), and one characterizing the steady-state PK of OROS and IR hydromorphone (Study B) (Moore et al. 2010). We sought to further characterize the single-dose and steady-state PK profiles of hydromorphone by evaluating time ≥50% *C*_*max*_ in healthy subjects after administration of oral OROS hydromorphone ER.

The current analysis was presented separately for both Study A (Richarz et al. 2011a) and Study B (Richarz et al. 2011b) in poster form at the American Pain Society 30th Annual Scientific Meeting, May 18–21, 2011, in Austin, TX.

## Methods

Detailed methods for each study have been published elsewhere (Moore et al. 2010; Moore et al. 2011).

### Participants

Study A (single dose, fed, or fasted) and Study B (steady state) were conducted according to the principles of the Declaration of Helsinki and current International Conference on Harmonization guidelines on Good Clinical Practice. Both study protocols were approved by an Institutional Review Board convening at MDS Pharma Services, Montréal, Québec, Canada. All subjects provided written informed consent.

Both studies enrolled men and women aged 18 to 55 years who were considered healthy, with a body mass index of 18 kg/m^2^ to 30 kg/m^2^ and body weight ≥50 kg. Subjects with a history of illness or current medical illness were excluded. Medication use (other than hormone replacement therapy, oral contraceptives, or acetaminophen) within 14 days prior to study treatment was prohibited. Subjects with a history or believed history of drug or alcohol abuse within the past 5 years were also excluded; a naloxone 0.8-mg injection was administered prior to study randomization and medication dosing to detect opioid dependence (Moore et al. 2010; Moore et al. 2011).

### Study design

#### Study A

Study A was a randomized, open-label, single-center, 3-period, crossover study. Following screening, subjects entered an open-label treatment phase consisting of 3 treatment periods, each 5 days in duration. All subjects received each of 3 treatments: a single oral dose of OROS hydromorphone ER 16 mg under fasting conditions (Treatment A); 4 oral doses of IR hydromorphone 4 mg every 6 hours over 24 hours under fasting conditions (Treatment B); and a single oral dose of OROS hydromorphone ER 16 mg under fed conditions (Treatment C, given immediately following the completion of a high-fat meal). Subjects were randomly assigned by a computer-generated schedule to receive each of the 3 treatments in 1 of 6 possible sequences (Additional file 1: Figure S1a) (Moore et al. 2011). Each treatment period was separated by a washout period of ≥7 and ≤14 days after dosing; given the approximate 13- to 15-hour apparent elimination half-life (*t*_*1/2,λ*_) of OROS hydromorphone ER, a maximum washout period of 2 weeks was thought to be sufficient to avoid any carryover effect that might influence analyses (Moore et al. 2011; Gupta and Sathyan 2007).

Subjects treated under fasting conditions fasted for ≥12 hours before dosing, whereas subjects treated under a fed state received dosing immediately following the completion of a high-fat meal. During each period, all subjects received a concomitant dosing regimen of naltrexone 50-mg oral tablets to counter the opioid effects of hydromorphone (7 single doses, 14 and 2 hours before the study drug dose and every 12 hours thereafter, up to 58 hours post-study drug dose) (Moore et al. 2011).

#### Study B

Study B was a randomized, open-label, single-center, multidose, 2-period, crossover study (Additional file 1: Figure S1b) (Moore et al. 2010). Subjects were randomly assigned to one of 2 possible treatment sequence groups and received each of the following treatments: 16 mg OROS hydromorphone ER administered orally once daily for 5 days and 4 mg IR hydromorphone administered orally every 6 hours for 5 days. Treatment periods were separated by a washout period of between 7 and 14 days after dosing. To block the subjective effects of hydromorphone, naltrexone 50 mg was given 14 and 2 hours before the first dose, and every 12 hours until 130 hours after initial dosing (Moore et al. 2010).

### Pharmacokinetic sampling and analysis

#### Study A

The PK sampling is explained in detail elsewhere (Moore et al. 2011). Briefly, venous blood samples for measurement of plasma hydromorphone concentrations were collected before dosing and at regular intervals after dosing until 72 hours post-dose. Concentrations of hydromorphone in plasma were measured by high-performance liquid chromatography (HPLC)–tandem mass spectrometry. The following PK parameters were estimated from the plasma data: *AUC* from time 0 to time of the last quantifiable concentration (*AUC*_*last*_); *AUC* from time 0 to over 24 hours (*AUC*_*0-24*_); the *AUC* extrapolated to infinity (*AUC*_*∞*_); *C*_*max*_; time to *C*_*max*_ (*t*_*max*_); *t*_*1/2,λ*_; and the first-order elimination rate constant (*λ*_*z*_) (Moore et al. 2011).

#### Study B

Venous blood samples for measurement of plasma hydromorphone concentrations were collected before dosing and at regular intervals after dosing until 120 hours postdose; further sampling between 96 and 120 hours was undertaken to characterize the steady-state profile of each formulation. Similar to Study A, hydromorphone plasma concentrations were measured by means of a validated and specific HPLC–tandem mass spectrometry technique, with a range of 0.05 ng/mL to 10.0 ng/mL. Primary PK analyses included *AUC*_*0-24*_; maximum plasma concentration at steady state (*C*_*maxss*_); time to *C*_*maxss*_ (*t*_*maxss*_); trough plasma concentration at steady state (*C*_*minss*_); and degree of fluctuation. Percentage fluctuations in hydromorphone concentrations (flux) were calculated from the formula ((*C*_*maxss*_ – *C*_*minss*_)/*C*_*ssav*_) × 100, where *C*_*ssav*_ is calculated as the ratio of *AUC*_*0-24*_ to the dosing interval *τ* (24 hours) (Moore et al. 2010).

### Safety evaluation

Safety in each study was evaluated by monitoring adverse events (AEs), physical examination, vital signs, clinical laboratory tests, and electrocardiographic monitoring. Continuous pulse oximetry was undertaken and respiration rate was monitored while patients were sleeping (Moore et al. 2010; Moore et al. 2011).

### Statistical analyses

For the present analyses, individual and mean plasma concentration-versus-time profiles were generated within each study. For each treatment, descriptive statistics were calculated for all PK parameters of hydromorphone.

A steady-state analysis using a mixed-effects analysis of variance (ANOVA) model with Helmert contrasts was performed to identify attainment of steady state in plasma hydromorphone concentrations. The model included treatment-sequence group, period, treatment, and time as fixed effects. Steady-state conditions were assumed if the Helmert contrasts were not significantly different on at least 3 predose values, assuming an *α*-level of 0.05. The total time spent at ≥50% *C*_*max*_ was calculated for both studies, using linear interpolation between the plasma concentration time points that crossed the 50% *C*_*max*_ threshold. Time above 50% of *C*_*max*_ was analyzed using a mixed-effects model with sequence, period, and treatment as fixed effects and subject-within-sequence as a random effect to account for the crossover design. The difference in least-square means between OROS hydromorphone ER (fasted and fed) and IR hydromorphone was deemed significant if *P* < 0.05.

The analyses included only data from participants who completed all PK assessments and was performed using the SAS^®^ (SAS Institute, Cary, NC, USA) MIXED and GLM procedures (Moore et al. 2010; Moore et al. 2011).

## Results

### Subject disposition and demographics

Thirty subjects were enrolled into each study. Fifty-nine subjects completed all treatment periods and were included in the PK analyses. One subject was withdrawn from Study B prior to dosing due to elevated creatine phosphokinase levels. The mean age of subjects in Study A was 42 ± 9 years, and 77% were male; mean age in Study B was 39 ± 8.5 years, and 50% were male. Full demographic characteristics of the subjects have been presented previously (Moore et al. 2010; Moore et al. 2011).

### Pharmacokinetic analysis

#### Concentration-time profiles

**Figure 1:**
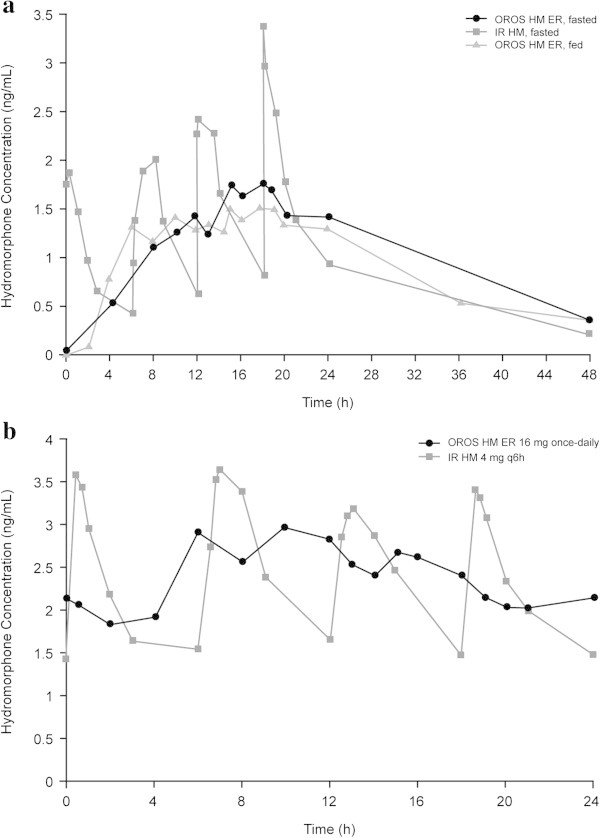
**Plasma hydromorphone concentration profiles for OROS hydromorphone ER and IR hydromorphone.** Panels show concentration profiles after single dosing **(a)** and at steady state **(b)**. ER, extended-release; IR, immediate-release.

In Study A, mean *AUC* comparisons of 16 mg OROS hydromorphone ER and IR hydromorphone 4 mg every 6 hours indicated bioequivalence in the fasted state (Table 1) (Moore et al. 2011). Mean *C*_*max*_ of IR hydromorphone in the initial 24-hour interval under fasted conditions was 89% and 100% higher than OROS hydromorphone ER under fasted and fed conditions, respectively (Table 1, Figure 1a) (Moore et al. 2011). Individual PK parameters for OROS hydromorphone ER were comparable under fed and fasted conditions (Table 1) (Moore et al. 2011).

**Table 1:** **Mean (SD) pharmacokinetic parameters after single dosing and at steady state for OROS hydromorphone ER and IR hydromorphone in healthy subjects**

Parameter, mean (SD)	OROS hydromorphone ER		IR Hydromorphone, fasted
	Fed	Fasted	
**Study A**			
No. of subjects	29	30	30
*AUC*_*last*_, ng•h/mL	45.9 (11.2)	46.9 (13.8)	43.9 (10.4)
*AUC*_*0-24*_, ng•h/mL	25.6 (6.5)	25.7 (6.6)	30 (7.6)
*C*_*max*_, ng/mL	1.8 (0.5)	1.9 (0.5)	3.6 (1.5)
*t*_*max*_, h^a^	16 (5.9–24.2)	17.9 (6.0–24.2)	18.5 (18.5–20.0)
*t*_*½,λ*_, h	14.4 (4.1)	14.4 (6.0)	12.7 (3.4)
Time ≥50% *C*_*max*_, h	23.6 (8.0)	21.6 (6.7)	5.9 (4.1)
**Study B**			
No. of subjects	29		30
*AUC*_*0-24*_, ng•h/mL	57.6 (16.3)		54.8 (14.8)
*C*_*maxss*_, ng/mL	3.54 (0.96)		5.28 (1.37)
*C*_*minss*_, ng/mL	2.15 (0.87)		1.47 (0.42)
*t*_*maxss*_, h^a^	11.9 (5.9–24.2)		7.0 (0.5–18.8)
Peak-to-trough fluctuation (flux), %	60.5 (41.1)		172 (57.6)
Time ≥50% *C*_*max*_, h	20.5 (4.1)		7.5 (4.8)

*AUC*_*0-24*,_ area under the concentration versus time curve from time 0 to 24 hours postdose; *AUC*_*last*_, area under the concentration versus time curve from time 0 to the last quantifiable concentration; *C*_*max*_, maximum plasma concentration; *C*_*maxss*_, maximum plasma concentration at steady state; *C*_*minss*_, trough plasma concentration at steady state; ER, extended-release; IR, immediate-release; *t*_*½,λ*_, apparent elimination half-life; *t*_*max*_, time to maximum plasma concentration; *t*_*maxss*_, time to maximum plasma concentration at steady state.

a. Median (range) reported for *t*_*max*_.

In Study B, 16 mg of OROS hydromorphone ER successfully maintained steady-state hydromorphone plasma concentrations within the same concentration range as 4 mg IR hydromorphone every 6 hours (administered at the same total daily dose) (Table 1) (Moore et al. 2010). Overall exposure to hydromorphone was similar with both treatments, as measured using *AUC*_*0-24*_ (Table 1) (Moore et al. 2010). The observed median *t*_*maxss*_ of OROS hydromorphone ER (11.9 hours) occurred approximately 5 hours after the *t*_*maxss*_ for IR hydromorphone (7.0 hours) (Table 1) (Moore et al. 2010).

At steady state, the mean (SD) degree of peak-to-trough fluctuation (flux) was 60.5% (41.1%) for OROS hydromorphone ER, compared with 172% (57.6%) with IR hydromorphone (Table 1) (Moore et al. 2010). Mean plasma hydromorphone concentrations over time for OROS hydromorphone ER and IR hydromorphone at steady state (day 5) are presented in Figure 1b (Moore et al. 2010).

#### Half-value duration (HVD)

**Figure 2:**
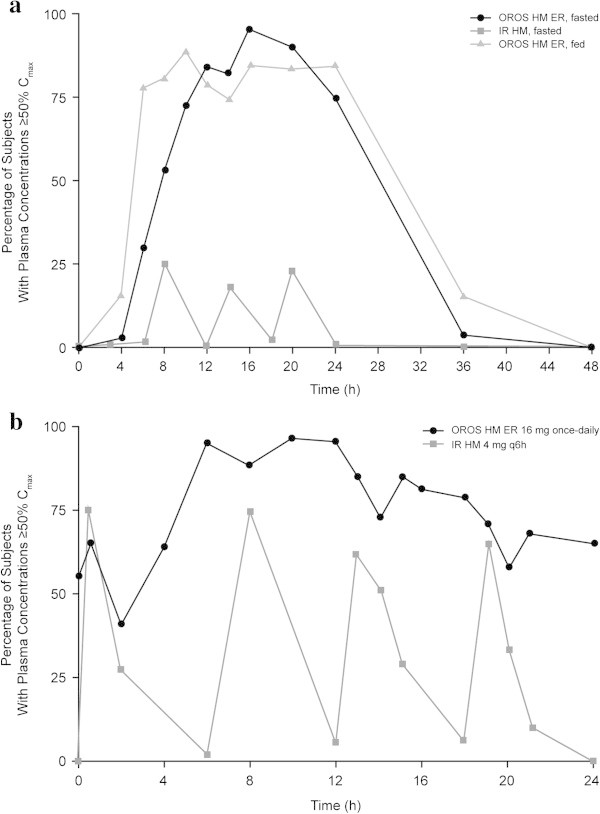
**Percentage of subjects with plasma hydromorphone concentrations ≥50%** ***Cmax*** **at each time point after dosing with OROS hydromorphone ER and IR hydromorphone.** Panels show percentages after single dosing **(a)** and at steady state **(b)**. *Cmax*, maximum plasma concentration; ER, extended-release; IR, immediate-release.

**Figure 3:**
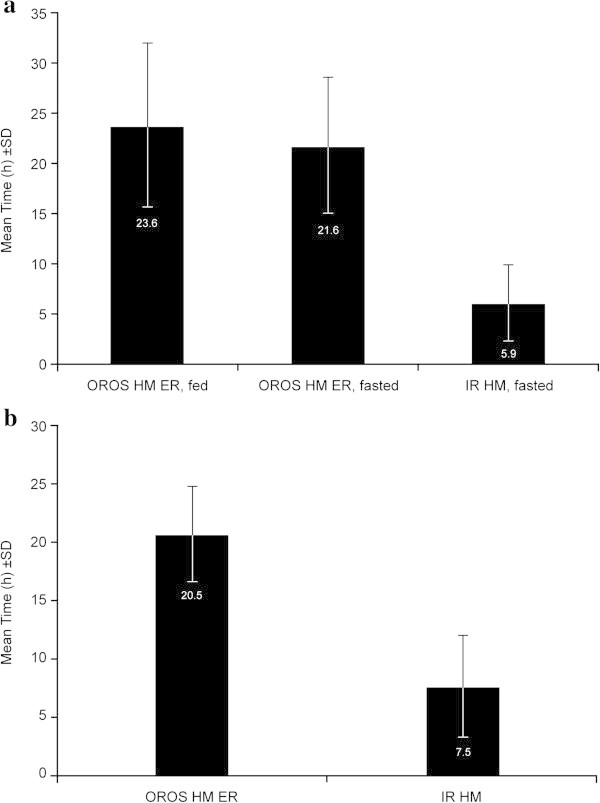
**Mean time spent ≥50%** ***Cmax*** **for OROS hydromorphone ER and IR hydromorphone.** Panels show mean time spent ≥50% *Cmax* after single doses of OROS hydromorphone ER and 4 doses of IR hydromorphone **(a)**, and at steady state **(b)**. *Cmax*, maximum plasma concentration; ER, extended-release; IR, immediate-release.

**Figure 4:**
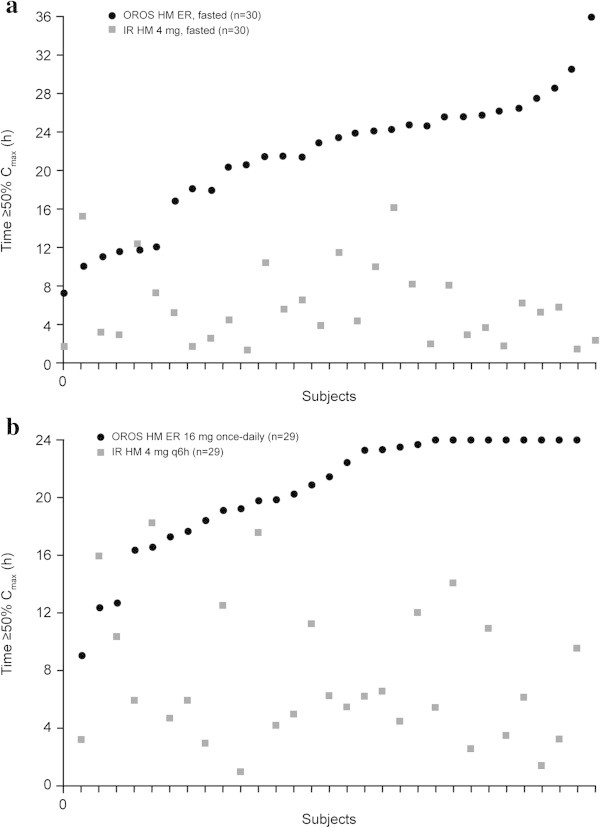
**Time spent ≥50%** ***Cmax*** **for OROS hydromorphone ER and IR hydromorphone by subject.** Panels show time spent ≥50% *Cmax* by subject after single dosing **(a)**, and at steady state **(b)**. *Cmax*, maximum plasma concentration; ER, extended-release; IR, immediate-release.

Compared with IR hydromorphone, a greater percentage of subjects receiving OROS hydromorphone ER had hydromorphone plasma concentrations ≥50% *C*_*max*_ at each time point after dosing in Study A (Figure 2a) and at all time points from 2 hours after dosing in Study B (Figure 2b). Based on individual subject data, a single 16-mg dose of OROS hydromorphone ER sustained plasma concentrations ≥50% *C*_*max*_ for a mean (SD) of 23.6 (8.0) hours and 21.6 (6.7) hours under fed and fasted conditions, respectively, compared with 5.9 (4.1) hours after a 16-mg total daily dose (given as 4 × 4-mg doses) of IR hydromorphone (*P* < 0.0001 for each OROS vs. IR comparison; Table 1 and Figure 3a) (Moore et al. 2010; Moore et al. 2011). Plasma hydromorphone concentrations remained ≥50% *C*_*max*_ ≥20 hours in approximately 70.0% of subjects after a single 16-mg dose of OROS hydromorphone ER. This occurred in no subjects who received a 16-mg total daily dose of IR hydromorphone (Figure 4a). At steady state, the mean (SD) time spent ≥50% *C*_*max*_ was 20.5 (4.1) hours for OROS hydromorphone ER and 7.5 (4.8) hours for IR hydromorphone (Table 1 and Figure 3b) (Moore et al. 2010; Moore et al. 2011). The time spent ≥50% *C*_*max*_ at steady state ranged from 9.1 to 24.0 hours (where it was capped) for the OROS hydromorphone ER and from 1.0 to 18.2 hours for IR hydromorphone (4 doses per day). Steady-state hydromorphone plasma concentrations remained at ≥50% *C*_*max*_ for ≥20 hours in 58.6% of subjects receiving OROS hydromorphone ER, compared with no subjects receiving IR hydromorphone at the same total daily dose (Figure 4b). Steady-state plasma concentrations remained ≥50% *C*_*max*_ for ≥16 hours in 89.7% and 6.9% of subjects, respectively (Figure 4b) (Moore et al. 2010; Moore et al. 2011).

### Safety

#### Study A

A total of 46 mild to moderate AEs were reported by 17 subjects (56.7%). Seventeen (37%) AEs were reported with OROS hydromorphone ER fasted, 21 (46%) AEs were reported with OROS hydromorphone ER fed, and 8 (17%) AEs were reported with IR hydromorphone fasted. Overall, the highest incidences of AEs were headache, dizziness, and constipation (Table 2) (Moore et al. 2011).

**Table 2:** **Incidence of treatment-emergent adverse events occurring in ≥1 patient overall in each study**

Adverse event, n (%)	OROS hydromorphone ER		IR hydromorphone, fasted (n = 30)
	Fed (n = 29)	Fasted (n = 30)	
**Study A**			
Headache	2 (7)	3 (10)	1 (3)
Dizziness	1 (3)	1 (3)	1 (3)
Constipation	1 (3)	1 (3)	1 (3)
Abdominal pain, upper	1 (3)	1 (3)	0 (0)
Nausea	2 (7)	0 (0)	0 (0)
Vomiting	1 (3)	1 (3)	0 (0)
Hyperhidrosis	1 (3)	1 (3)	0 (0)
**Study B**	**OROS hydromorphone ER (n = 29)**		**IR hydromorphone (n = 30)**
Chest pain	2 (6.9)		2 (6.7)
Erythema	2 (6.9)		1 (3.3)
Pruritus	2 (6.9)		1 (3.3)
Constipation	1 (3.4)		1 (3.3)
Fatigue	1 (3.4)		1 (3.3)
Headache	1 (3.4)		1 (3.3)
Somnolence	1 (3.4)		1 (3.3)

ER, extended-release; IR, immediate-release.

#### Study B

Overall, 21 AEs were reported by 7 subjects (24%) receiving OROS hydromorphone ER and 16 AEs were reported by 10 subjects (34.5%) receiving IR hydromorphone. The most commonly reported treatment-emergent AEs occurring in ≥5% of subjects are presented in Table 2 (Moore et al. 2010). Chest pain occurred in 2 patients during the OROS and IR administration and each event was considered mild and unlikely to be related to study medication. Localized erythema occurred in 2 subjects during OROS administration and 1 subject during IR administration. All events were considered mild in severity; erythema was considered possibly related to study medication in only 1 subject (during OROS administration) (Moore et al. 2010).

Both OROS hydromorphone ER and IR hydromorphone, administered in conjunction with naltrexone to counter the opioid effects of hydromorphone, appeared to be generally safe and well tolerated, with no serious AEs occurring during either study (Moore et al. 2010; Moore et al. 2011).

## Discussion and conclusions

The current analysis demonstrated bioequivalence of once-daily OROS hydromorphone ER and 4-times-daily IR hydromorphone at equivalent total daily doses. *HVD*, which corresponds to the length of time that plasma concentrations are ≥50% *C*_*max*_, has been applied complementarily to conventional parameters as a measure of the performance of ER formulations (Steinijans 1990; Drewe et al. 1992; Meier et al. 1974). Despite the higher *C*_*max*_ with each 4-mg dose of IR hydromorphone compared with the OROS formulation, the time spent at ≥50% *C*_*max*_ for subjects receiving OROS hydromorphone ER was, on average, 2.7 times longer at steady state than for subjects receiving 4 doses of IR hydromorphone over a 24-hour period. These data are consistent with results reported by Angst et al. (2001), in which the mean (SD) duration of hydromorphone plasma concentration ≥50% *C*_*max*_ was 21.6 (8.1) hours in subjects following a single dose of 16 mg OROS hydromorphone ER (Angst et al. 2001).

In the current analysis, once-daily OROS hydromorphone ER tablets were shown to successfully maintain steady-state hydromorphone plasma concentrations by day 4 within the same concentration range as the 4-times-daily IR hydromorphone tablets (administered at the same total dose per day) but with less fluctuation. The mean degree of fluctuation for OROS hydromorphone ER was 65% lower than that observed with IR hydromorphone (Moore et al. 2010). These data also demonstrate that mean steady-state once-daily OROS hydromorphone ER concentrations are elevated for most of the 24-hour dosing period and for significantly longer than with the IR hydromorphone dosing regimen at the same total daily dose, due to the gradual release of hydromorphone from the tablet and ongoing enterohepatic circulation of the drug over the dosing period.

Although OROS hydromorphone ER and IR hydromorphone had comparable AUC data, the ER formulation—with its extended period of time during which plasma concentrations are ≥50% *C*_*max*_—may be beneficial in patients with chronic pain who require around-the-clock opioid therapy. Although the parameters presented do not necessarily imply analgesia per se, they do support a consistent performance for this formulation over time.

When administered for up to 5 consecutive days, both OROS hydromorphone ER and IR hydromorphone appeared to be well tolerated in healthy subjects. It should be noted that, as is common in PK studies of this nature, naltrexone was administered to reduce the likelihood of opioid-related AEs. Naltrexone could potentially alter the PK profile of hydromorphone, because it reverses the slowing of gastric transit associated with opioids (Murphy et al. 1997). This is a potential limitation of this study, although it should be acknowledged that all treatment groups received naltrexone. Earlier work showed that, although coadministration of OROS hydromorphone with naltrexone under fasting conditions resulted in a 39% increase in *C*_*max*_, this was not accompanied by significant changes in *t*_*max*_; AUC from time zero to τ (AUC_0-τ_), where τ is the last 24-hour dosing interval; or AUC_∞_ (Sathyan et al. 2007). The PK results from the current studies are consistent with those reported by Angst et al. (2001), in which naltrexone was not used in conjunction with hydromorphone (Angst et al. 2001).

The post-hoc nature of the analysis is another limitation of the study. These studies were not specifically designed to correlate PK measures with analgesia; therefore, no conclusions about *HVD* as a predictor of duration of efficacy can be drawn. However, the *HVD* (and flux) offer information about the stability of plasma concentrations over time with ER analgesics, which may be important in populations receiving multiple doses of analgesics over a prolonged period of time.

Additional studies will be necessary to fully evaluate the relationship of the time spent ≥50% *C*_*max*_ to duration of analgesic effect associated with OROS hydromorphone ER.

Additional prospective studies in chronic pain sufferers may help verify the extent to which *HVD* or other plateau times can differentiate the profile of ER versus IR opioids, and the degree to which such differences are associated with sustained analgesia and other functional improvements.

### Ethical standards statement

The 2 studies reported on in this paper conformed to the laws of the United States and Canada.

## Electronic supplementary material

Additional file 1: Figure S1: Study periods and treatments. Participants in Study A were randomly assigned to one of 6 possible treatment sequences. Treatment A consisted of single doses of OROS hydromorphone ER 16 mg administered under fasted conditions; Treatment B consisted of IR hydromorphone 16 mg (4 mg every 6 hours for 24 hours) under fasted conditions; Treatment C consisted of single doses of OROS hydromorphone ER 16 mg immediately following the completion of a standard high-fat breakfast (approximately 1000 kcal, of which 500 to 600 kcal was derived from fat) (a). Participants in Study B were randomly assigned to receive either OROS hydromorphone ER 16 mg or IR hydromorphone 16 mg (total daily dose) for 5 sequential days each in one of two sequences (b). ER, extended-release; IR, immediate-release. (JPEG 349 KB)
