# Supplementary figures and images for: Complementary pharmacokinetic measures to further define the profile of once-daily OROS hydromorphone ER during single-dose and steady-state dosing
Source: Springerplus. 2013 Nov 21;2:625. doi: 10.1186/2193-1801-2-625 (PMC3863398; doi:10.1186/2193-1801-2-625)

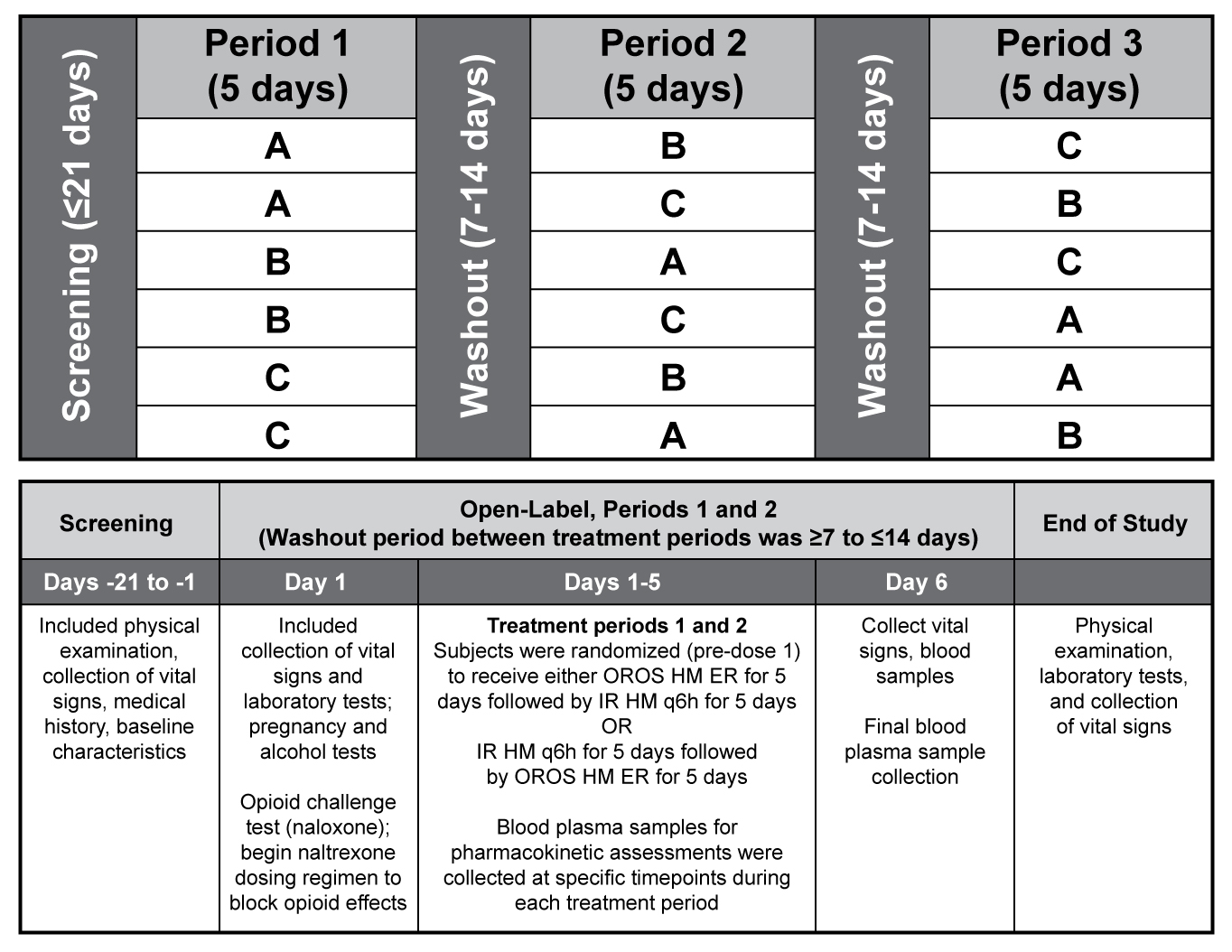

Supplement: Supplementary file 1 — Additional file 1: Figure S1: Study periods and treatments. Participants in Study A were randomly assigned to one of 6 possible treatment sequences. Treatment A consisted of single doses of OROS hydromorphone ER 16 mg administered under fasted conditions; Treatment B consisted of IR hydromorphone 16 mg (4 mg every 6 hours for 24 hours) under fasted conditions; Treatment C consisted of single doses of OROS hydromorphone ER 16 mg immediately following the completion of a standard high-fat breakfast (approximately 1000 kcal, of which 500 to 600 kcal was derived from fat) (a). Participants in Study B were randomly assigned to receive either OROS hydromorphone ER 16 mg or IR hydromorphone 16 mg (total daily dose) for 5 sequential days each in one of two sequences (b). ER, extended-release; IR, immediate-release. (JPEG 349 KB) [file 40064_2013_703_MOESM1_ESM.jpeg]
